# Emergency Physicians Think in Spirals

**DOI:** 10.7759/cureus.381

**Published:** 2015-11-17

**Authors:** Tia Renouf, Desmond Whalen, Megan Pollard, Adam Dubrowski

**Affiliations:** 1 Emergency Medicine, Memorial University of Newfoundland; 2 Clinical Learning and Simulation Centre, Memorial University of Newfoundland; 3 Emergency Medicine, Pediatrics, Memorial University of Newfoundland; 4 Marine Institute, Memorial University of Newfoundland

**Keywords:** emergency medicine, rapid, snapps, spiral, clinical reasoning, simulation

## Abstract

As adult learners, junior clerks on core rotations in emergency medicine (EM) are expected to “own” their patients and follow them from presentation to disposition in the Emergency Department (ED). Traditionally, we teach clerks to present an exhaustive linear list of symptoms and signs to their preceptors. This does not apply well to the fast-paced ED setting. Mnemonics have been developed to teach clerks how to present succinctly and cohesively. To address the need for continual patient reassessment throughout the patient’s journey in the ED, we propose a complimentary approach called SPIRAL.

## Introduction

Adult learning theory suggests that clerks are internally motivated, self-directed, and active learners who appreciate practical outcomes. In addition, teaching may be best when students’ learning style preferences are considered [1]. Since many clerks belong to Generation Y, a group that characteristically has active learning style preferences, it follows that a busy ED is a good place for them to acquire skills knowledge and attitudes [2]. Continual re-evaluation is an approach that needs to be taught for complete and safe patient care.

The ED is a unique teaching environment. Cognitive load is high and timely patient interventions are often critical. Lack of follow-through can lead to medical error. The exhaustive traditional presentations taught to clerks are impractical in the ED. They may omit important information that is essential to comprehensive and timely ED patient management. Mnemonics techniques known as RAPID (Resuscitation, Analgesia and assessment, Patient needs, Interventions, Disposition) [3] and SNAPPS (Summarize history and findings, Narrow the differential; Analyze the differential; Probe preceptor about uncertainties; Plan management; Select case-related issues for self-study) have been developed as prompts for clinical clerks to present cases concisely yet thoroughly, but they are linear and exist at one given point in time [4]. This is far from the typical ED learning opportunities.

**Figure 1:**
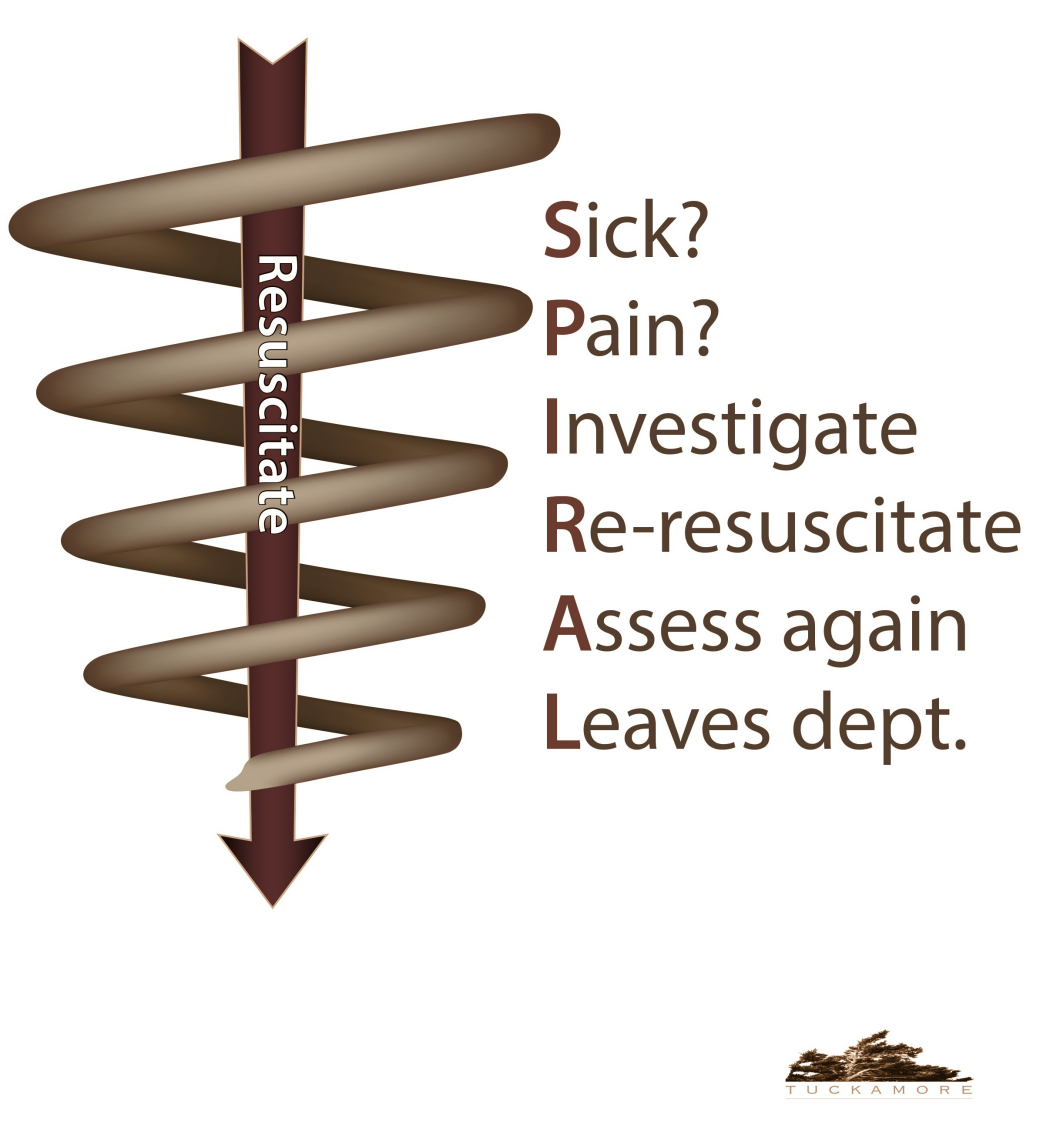
Spiral Approach to Clinical Reasoning in the Emergency Room Resuscitation is at the centre of the spiral and all interventions must circle back to it. (Image created by Luke Merdsoy)

In order for the clerks to “own” their patients and follow them over their entire ED course, we need to teach them to modify their linear thought patterns into spirals in which they always re-evaluate priorities and circle back to critical problems while re-assessing resuscitation efforts, interventions, and disposition plans. In their initial presentations to their preceptors, and as prompted with RAPID, clerks should include important details, such as the patient’s social situation and why they came to the ED today, but these issues are less likely to need continual re-evaluation throughout the patient’s ED stay. For this reason, the SPIRAL (Figure 1) is broad where the patient enters the ED, and narrows over time as the patient approaches ED disposition. We developed the SPIRAL mnemonic as a prompt for clerks. It may be used in conjunction with RAPID and SNAPPS.

Simulation is an effective tool for teaching clinical reasoning. Deliberate practice occurs when a learner tries to improve performance with each practice attempt by using repetitive performance, expert assessment, and feedback. Planning, concentration, repetition, and reflection modify the deliberate practice technique for the acquisition of clinical skills [5]. This is relevant for clerks who are learning the SPIRAL approach for the first time. Simulation provides opportunities for presenting a specific and well-structured interaction with a patient. This is essential to the learning process, yet impossible to achieve in the ED. For example, optimal challenge framework speculates that novice learners should start with an easy version of a task or a scenario, and as their knowledge and skill levels advance, the difficulty of the simulation should also increase. This is also known as sequential or progressive simulation [6-7], and it can be used to re-create the ED environment to optimize learning.

Sequential simulation [8] may also be an effective tool for teaching SPIRAL. It has been used to teach and engage varied health practitioners and a patient’s family in, for example, a young asthmatic’s journey through the health care system while she has an acute attack. SPIRAL involves several patient re-assessments both over time and under evolving clinical circumstances. Sequential scenarios – moving, in our case, from the low acuity ED space to a monitored bed and then resuscitation area, and involving at different intervals the preceptor, nursing staff, and ICU/surgical consultants - may apply well to such a fluid situation.

ED rotations may be the clerk’s first exposure to following and continually re-evaluating potentially ill, undifferentiated patients over a relatively short period of time. We use the deteriorating patient scenario (DPS) [9] along with sequential simulation because both are well-suited to the nimble course corrections that the learner must make in this case.

## Technical report

We present a case of biliary sepsis caused by an impacted common bile duct stone. The patient presents with abdominal pain, jaundice, and low-grade fever. Although she is initially fairly well, the fever and jaundice portend deterioration over time and mandate frequent re-evaluation and resuscitation. Initially, the learner presents a focused and complete ED history and physical, formulates a differential diagnosis starting with the most likely disease process, and, importantly, includes the most potentially serious “can’t miss” diagnoses. Resuscitation and analgesia are discussed along with potential disposition plans. Social circumstances and reasons that the patient came to the ED today are included. After this initial case presentation, the learner must “own” their patient by continually re-assessing her; if they do not, prompts or hints are given. If the prompts are not followed, the patient deteriorates. In order to achieve execution of all skills, procedures, and protocols, we use hybrid simulation, which refers to the simultaneous use of several simulation resources. We use a standardized patient (SP) in tandem with monitors and task trainers for intravenous access, as the learner will need to read subtle body language cues to decide whether the patient is sick or in pain.

Three separate sequential simulations will flow into one another. In the first setting, the patient is stable, lying without monitors in a low-acuity ED bed. The scenario moves to a monitored mid-level acuity bed as the patient deteriorates. Finally, ICU and surgical consultants are in attendance in the resuscitation suite.

While an SP plays the patient, the physicians and nurses involved in this case will play themselves. The simulation for the latter group is unscripted to enhance realism [8]. 

### Learners

The learners in this scenario are junior clerks who are expected to learn how to follow their patients from presentation in the ED until discharge. While these clerks may have learned how to follow inpatients over days or weeks, the undifferentiated ED patient's journey is much shorter and less predictable. The SPIRAL mnemonic is used to teach the concept of spiral thinking. This approach is used for continual patient reassessment and investigation that revolves around the ever-present core need for resuscitation and the patient’s response to it.

### Pre-briefing

The learners are told they will initially present a patient with undifferentiated abdominal pain to their ED preceptor. They must then continually reassess the patient from arrival in the ED to disposition and tell the preceptor of significant developments in the case. They are taught the SPIRAL mnemonic, which is a prompt to continually evaluate the patient to decide if she is sick, in pain, merits further investigations, or needs repeat resuscitation and re-assessment throughout her course in the ED. The key is anticipating deterioration while remembering to check the patient continually and revising resuscitation efforts, as well as recognizing the need for new investigations and a disposition plan. Every learner is also handed a SPIRAL cure card that they are asked to keep with them during the simulation. They are allowed to use the cue card at any point if they need to refresh what the acronym stands for, as described in Figure 1.

**Figure 2:**
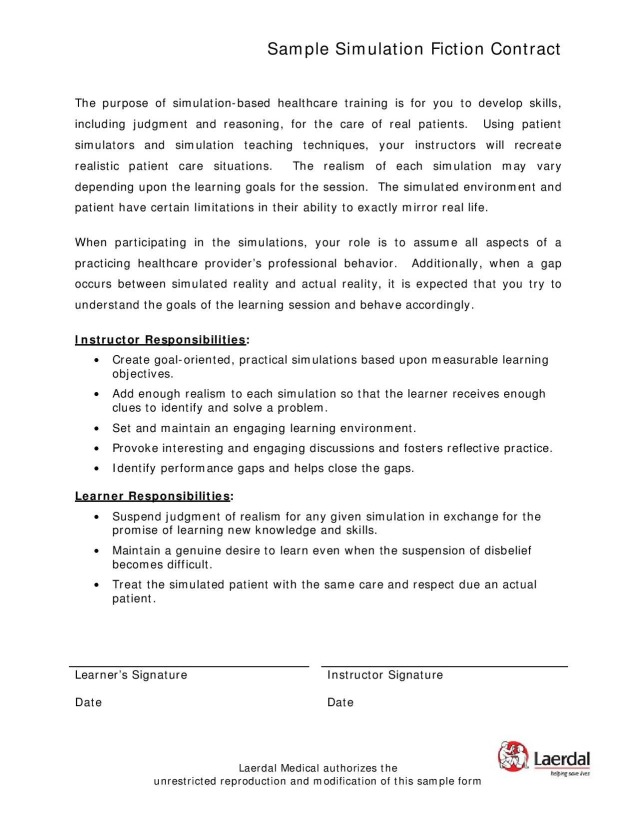
Simulation Fiction Contract

The Laerdal fictional contract (Figure 2) is discussed, in which learners acknowledge that while the scenario is not real, they will proceed as though it is. Any equipment limitations are noted, and it is explained that the exercise is strictly formative.

### Case

The learning objectives for this case (Table 1) are:

1. Learn to present a case concisely, yet completely;

2. Anticipate potential deterioration and manage the patient over time using SPIRAL;

3. Learn to manage biliary sepsis.

**Table 1:** A Stepwise, Detailed Scenario Template

Scenario		
You are a clinical clerk in a busy tertiary care ED and you are ready to present your case to your ED preceptor. You are informed that you are expected to follow your patient throughout their entire ED stay.		
Begin scenario - learner enters the patients room		
Objective 1: Learning to present case concisely and completely		
Additional Scenario Data/ History and Physical Findings	Vital Signs	Appropriate Learner Action
Patient is in lying in bed in a non-monitored room, complaining of abdominal pain. The patient does not look sick but is in pain. Has had sharp RUQ pain radiating to the back intermittently for a week. Pain lasts one hour. Worse with eating fat and seems to stop on its own, but returns. The pain is so bad it makes her vomit. She is slightly jaundiced. Abdomen is tender in RUQ with positive Murphy’s sign. Was in the ED with this yesterday and is waiting for an outpatient ultrasound. Lives at home with husband. Otherwise well, on no meds, has no allergies, and has no significant PMH. She came to ED today because of vomiting and unbearable pain. If the symptoms settle and the patient remains well, expect discharge with US the next day. If not, US should be done today and general surgery consulted.	Vital signs: BP 120/80, HR 102, T 37^5^, RR 18 GCS 15	Takes initial history, performs physical. Considers analgesia. Orders appropriate bloodwork Presents focused history and physical, appropriate differential diagnosis starting with the most likely. Considers “cannot miss” diagnoses. Remembers to comment on why the patient comes in today and relevant social considerations. Considers a disposition plan.
Objective 2: Learning to manage the patient and SPIRAL over time		
Additional Scenario Data/ Physical Exam Findings	Vital Signs	Appropriate Learner Action
If learner checks, 20 minutes after the patient is given analgesia, pain is less. PROMPT: If the learner does not check, patient’s pain gets much worse and the nurse prompts to reassess the patient. If examined: still slightly tender RUQ. The patient looks slightly unwell	Stable	Learner remembers to check on patient Checks bloodwork and only the CBC is ready.
Laboratory Results		
If ordered: Hgb 132, WBC 18, 82% neutrophils. Plats 150 ECG: Figure 1		
40 minutes after presentation the learner must check on the patient		
If learner checks, patient’s pain is back, worse than ever. If learner does not check, patient will deteriorate. PROMPT: nurse will prompt learner to re-assess patient, saying the pain is back. She is vomiting. She looks unwell and in pain. Abdomen is soft but very tender RUQ.	Vital Signs: BP 150/92, HR 127, T 38.5, RR 24 GCS15	Checks on outstanding bloodwork, notes fever and HR and that the pain is hard to control. Orders fluid, blood cultures and US stat.
Gives another dose of analgesia	Vital signs same	
Laboratory results		
LFT and amylase abnormal		
		Leaves message in OR for general surgery
Laboratory/ECG/Diagnostic imaging results		
Ultrasound shows impacted stone in GB neck, sludge, thick gallbladder wall, and much stranding.		
60 minutes after presentation learner checks on the patient. If the learner does not check, the patient deteriorates further.		
The patient now looks very sick. The pain has not settled. The RUQ is very tender.	Vital signs: BP 80/60, HR140, T 39, RR 22 GCS 15	Learner considers pros and cons of more analgesia and diagnoses stone, possible biliary sepsis. Orders blood cultures, lactate and VBG, fluid bolus, second iv and appropriate antibiotics. Calls surgery stat and ICU. Moves the patient to a monitored bed.
Laboratory/ECG/Diagnostic imaging results		
Lab results as ordered by learner: Lactate 3.5, VGB normal.		
Objective 3: Learning appropriate disposition in the ED		
80 minutes after presentation, learner checks the patient. Looks somewhat better, but still very unwell. Pain is less.	Vital signs: BP 90 HR 118, T 38^5^, RR 26 Vital signs same	Gen Surgery and ICU at the bedside and arranging admission/OR
Appropriate treatment results in the simulated patient stabilizing		
End scenario		

### Debriefing

Following the scenario, the students are encouraged to discuss openly how they felt about the simulation. They learn by reflecting on their experiences. We use the frame discovery model [10-11] for debriefing, an approach that allows learners to make mistakes without being judged. It is assumed that learners make decisions for particular reasons that must be understood within their own frames of reference in order for incorrect choices to be modified. The teacher/learner ratio will not exceed 1:1 as it may be daunting for junior learners to discuss the learning gaps that emerge during the simulation. 

## Discussion

Biliary colic is a common ED presentation. Patients are predominantly female and in their reproductive years. While they often have significant pain, these patients are usually anicteric, afebrile, and generally well. They typically have right upper quadrant (RUQ)tenderness and a benign abdominal examination, but they can deteriorate over time and develop cholecystitis or biliary sepsis. This typically happens when the impacted gallstone fails to dislodge and intraluminal pressures rise inside the gallbladder. Ultrasound examination will usually show intrahepatic ductal dilation in biliary sepsis. While the presence of jaundice is most common in biliary sepsis and liver function tests are typically normal in biliary colic, these disease entities fall on a spectrum, so there is considerable overlap. Fever is always worrisome. The key in this exercise is for clerks to recognize “red flags” and anticipate deterioration while using the SPIRAL mnemonic to continually re-evaluate the patient and her resuscitative outcomes.

## Conclusions

The SPIRAL mnemonic may be a useful addition to RAPID and SNAPPS. It adds the dimension of time to the linear thought processes for clerks who are learning to follow their patients through their entire ED stay.
